# Recent progress in nanomedicine for enhanced cancer chemotherapy

**DOI:** 10.7150/thno.57828

**Published:** 2021-04-19

**Authors:** Guoqing Wei, Yu Wang, Guang Yang, Yi Wang, Rong Ju

**Affiliations:** 1 Chengdu Women's and Children's Central Hospital, School of Medicine, University of Electronic Science and Technology of China, Chengdu, 611731, PR China.; 2 College of Medicine, Southwest Jiaotong University, Chengdu, 610031, PR China.; 3 School of Life Science and Engineering, Southwest Jiaotong University, Chengdu, 610031, PR China.

**Keywords:** chemotherapy, combination therapy, nanocarriers, nanomedicine, cancer therapy

## Abstract

As one of the most important cancer treatment strategies, conventional chemotherapy has substantial side effects and leads easily to cancer treatment failure. Therefore, exploring and developing more efficient methods to enhance cancer chemotherapy is an urgently important problem that must be solved. With the development of nanotechnology, nanomedicine has showed a good application prospect in improving cancer chemotherapy. In this review, we aim to present a discussion on the significant research progress in nanomedicine for enhanced cancer chemotherapy. First, increased enrichment of drugs in tumor tissues relying on different targeting ligands and promoting tissue penetration are summarized. Second, specific subcellular organelle-targeted chemotherapy is discussed. Next, different combinational strategies to reverse multidrug resistance (MDR) and improve the effective intracellular concentration of therapeutics are discussed. Furthermore, the advantages of combination therapy for cancer treatment are emphasized. Finally, we discuss the major problems facing therapeutic nanomedicine for cancer chemotherapy, and propose possible future directions in this field.

## Introduction

Conventional chemotherapy is a crucial component of cancer treatments for various cancer types, and the treatment strategy is to use toxic drugs to kill cancer cells 1, 2. Many chemotherapeutic drugs have been discovered or synthesized since World War II 3. Although there have been great breakthroughs in cancer treatment, cancer remains a major life-threatening disease worldwide. For instance, 18.1 million new cancer cases, and 9.6 million cancer deaths occurred in 2018 4. At present, the low accumulation/retention of drugs in the tumor is acknowledged as a factor leading to the failure of clinical chemotherapy against cancer. Furthermore, chemotherapy usually induces multidrug resistance (MDR), which refers to a resistance phenotype, and cancer cells become resistant to different drugs with varying structures and molecular resemblances 5. Therefore, exploring and developing more efficient and simpler cancer treatment methods have important research significance and clinical value.

In recent years, nanocarrier-based drug delivery systems (NDDSs) (*e.g.,* polymeric micelles, liposomes, and organic/inorganic nanoparticles) have attracted substantial interest in cancer therapeutics because of their special physical and chemical properties 6, 7. In contrast to anticancer drugs without carriers, NDDSs can deliver higher doses of drug to tumor tissue *via* enhanced permeability and retention (EPR) effects and decrease the adverse effects of high doses 8. To date, many nanoformulations have been approved for clinical applications in cancer chemotherapy, and several nanomedicines are undergoing clinical trials (see Table 1). To further utilize the advantages of NDDSs, researchers have been exploring and fabricating many functionalized NDDSs by 1) modifying the surface of nanocarriers with targeting ligands on to improve their enrichment in tumor tissues 26 and 2) endowing NDDSs with specific responsiveness for drug release (pH 27, enzymes 28, glutathione (GSH) 29 and temperature 30) *via in vivo* and *in vitro* stimulation. Multifunctional NDDSs have shown good prospects in solving the problems of low drug delivery efficiency and unsatisfactory anticancer effects (especially for treatment of MDR tumors), laying a foundation for application of NDDSs in clinical practice. In addition, due to the limitations of single chemotherapy regiments, combined treatment strategies based on NDDSs are also emerging 31, 32.

**Figure 1:**
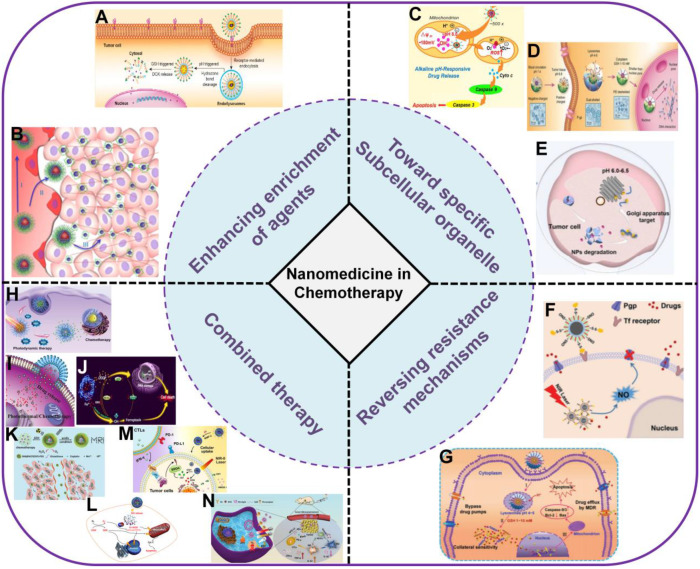
Schematic illustration of nanocarrier-based drug delivery systems (NDDS) for improving cancer chemotherapy based on different strategies. (A) Targeted drug delivery 45. Copyright 2016, ACS Publications, (B) Promoting tissue penetration 59. Copyright 2016, ACS Publications, (C) Mitochondria-targeted chemotherapy 74. Copyright 2018, Elsevier, (D) Nucleus-targeted chemotherapy 78. Copyright 2015, Wiley-VCH, (E) Golgi-targeted chemotherapy 80. Copyright 2019, ACS Publications, (F) Inhibition of P-gp 97. Copyright 2017, Wiley-VCH, (G) π-π stacked dual anticancer drug 98. Copyright 2016, Wiley-VCH, (H) Combination with PDT 136. Copyright 2020, Wiley-VCH, (I) Combination with PTT 142. Copyright 2020, Wiley-VCH, (J) Combination with CDT 113. Copyright 2020, ACS Publications, (K) Combination with radiotherapy 114. Copyright 2017, Wiley-VCH, (L) Combination with gas therapy 163. Copyright 2018, ACS Publications, (M) Combination with immunotherapy 166. Copyright 2021, Elsevier, (N) Multiple combination therapy 133. Copyright 2019, Wiley-VCH.

In this review, we aim to present a discussion on the significant research progress in improving cancer chemotherapy based on nanomedicine (Figure 1). This review is generally divided into five parts. In the first part, methods to increase enrichment of drugs in tumor tissues relying on different targeting ligands (targeting tumor blood vessels or cell membranes) and promoting tissue penetration are summarized. In the second part, specific subcellular organelle-targeted chemotherapy is discussed. In the third part, different combinational strategies to reverse MDR and improve the effective intracellular concentration of therapeutics are discussed. In the fourth part, the advantages of combination therapy (*e.g.,* chemotherapy combined with phototherapy, chemodynamic therapy, gas therapy, immunotherapy and multiple therapies) for cancer treatment are emphasized. Finally, we discuss the major problems therapeutic nanomedicine facing in cancer chemotherapy, and propose possible future directions in this field.

## Enhancing enrichment of chemotherapy agents in tumor tissue

### Targeted drug delivery

The ultimate goal of NDDSs is to achieve targeted drug therapy. In the past decade, to improve drug delivery efficiency, NDDSs with active targeting functions has become a heavily researched topic. To deliver more drugs to tumor tissue/cells, NDDSs can be modified with different targeting ligands on their surfaces, which allow them to specifically target tumor blood vessels or tumor cells.

#### Targeting tumor blood vessels

In the rapid growth process of tumor tissue, specific antigens or receptors are abnormally expressed on the surface of tumor vascular endothelial cells, while they are less or even unexpressed on the surface of blood vessels in normal tissues 33, 34. Therefore, researchers have grafted corresponding antibodies or ligands onto the surface of NDDSs to increase their enrichment in tumor blood vessels and achieve targeted delivery of drugs.

Because arginine/glycine/aspartic acid (RGD) can specifically bind to the integrin receptor *α_v_β_3_*, which is overexpressed in tumor neovascularization 35-37, it is often used as a tumor vascular targeting ligand and used to modify on the surface of nanocarriers to achieve tumor-targeted therapy. For example, Schiffelers et al. 38 reported liposomes with cyclic 5mr RGD (c(RGDf(ε-S-acetyl-thioacetyl)K) peptides that could target integrin *α_v_β_3_* on tumor vascular endothelial cells. The results of *in vitro* experiments confirmed that the modified liposomes significantly increased drug accumulation in tumor tissues compared to the non-RGD-modified liposomes. In addition, endothelial growth factor receptor 1 (VEGFR-1/Flt-1) and receptor 3 (VEGFR-3) are highly expressed on various tumor vascular endothelial cells. Therefore, VEGFR-1 and VEGFR-3 are also used as targeting ligands for promoter-targeted delivery. For example, Wang et al. 39 reported new tumor blood vessel-targeting nanoparticles, vincristine-loaded and F56-peptide conjugated nanoparticles (named F56-VCR-NPs); the F56 peptide has high affinity and specific VEGFR-1 binding ability and can achieve a high degree of cell internalization. *In vitro* and *in vivo* experimental results confirmed that F56-VCR-NP accurately targeted neovascularization in colorectal cancer, inducing tumor vascular endothelial cells to internalize nanoparticles, and significantly prolonged the survival time of mice without significant toxicity. The Esbp peptide (DITWDQLWDLMK) can also be used as a vascular targeting ligand due to its high affinity for E-selectin 40. For example, Shamay et al. 41 synthesized N-(2-hydroxypropyl) methacrylamide (HPMA) copolymers conjugated to Esbp peptide and equipped with doxorubicin (DOX) (P-(Esbp)-DOX). The results of *in vivo* experiments showed that P-(Esbp)-DOX significantly reduced tumor growth rate and prolonged the survival rate of lung cancer mice compared to those treated with a copolymer (P-DOX) or free DOX. In addition to the vascular targeting ligands mentioned above, KDEPQRRSARLSAKPAPPKPEPKPKKAPAKK peptide (F3), a 31-amino acid peptide, can also be used as a tumor-targeting peptide due to its preferential targeting of tumor blood vessels and tumor cells 42 and is usually grafted onto the surface of nanoparticles to target the tumor vasculature and increase the accumulation of nanoparticles in tumor blood vessels.

#### Targeting tumor cell membranes

Compared with normal cells, certain specific receptors or antigens are overexpressed on tumor cell membranes. Therefore, surface modification of nanocarriers with cell membrane targeting ligands can endow them with active targeting capabilities. Cell membrane targeting ligands mainly include folic acid (FA) 43, hyaluronic acid (HA) 44, phenylboronic acid (PBA) 45, aptamers 46, and peptides 47, et al.

**Figure 2:**
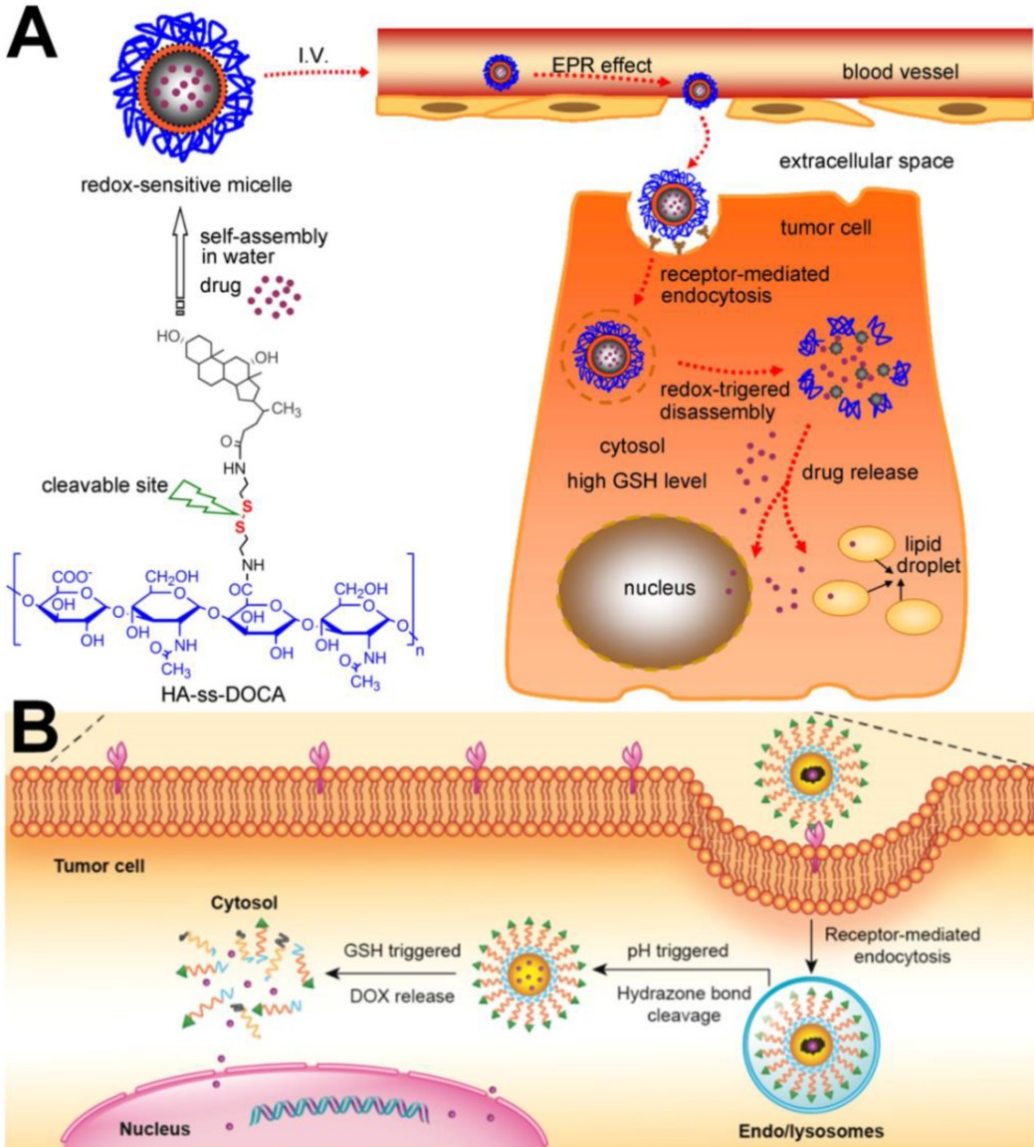
(A) Illustration of the self-assembly and intracellular trafficking pathway of redox-sensitive HA-ss-DOCA micelles 50. Copyright 2012, Elsevier. (B) PBA ligand-mediated endocytosis and Intracellular drug release triggered by GSH 45. Copyright 2016, ACS Publications.

As the most commonly used cell membrane targeting ligand, FA can bind to the folate receptor, which is overexpressed on the membrane surface of a variety of tumor cells (*e.g.,* breast cancer, ovarian cancer, and osteosarcoma cells). Zhou et al. 48 designed and developed actively targeted prodrug polymer micelles. First, FA was conjugated to the end of hydrophilic chain segments of the amphiphilic copolymer polyethylene glycol-*b*-polycaprolactone (PEG-*b*-PCL). DOX was then grafted on the end of the hydrophobic chain segments through an acid-sensitive bond, and FA-PEG-*b*-PCL-hyd-DOX micelles were finally prepared. Flow cytometry (FACS) and laser confocal microscopy (CLSM) confirmed that the FA-modified prodrug micelles could be internalized in a large amount by 4T1 tumor cells, and *in vivo* experimental studies showed that the FA-modified prodrug micelles could increase DOX enrichment in tumor tissues and exhibit better antitumor activity than micelles without FA modification. Similarly, HA, another commonly used targeting ligand, can specifically bind to the overexpressed CD44 receptor on the cell membrane and increase the cell membrane targeting ability of nanocarriers 49. For example, Li et al. 50 successfully developed a targeted cell drug delivery system based on redox sensitivity: hyaluronic acid-deoxycholic acid conjugate (HA-ss-DOCA) (Figure 2A). It was confirmed that HA-ss-DOCA micelles could be internalized in a large amount by human breast adenocarcinoma cells (MDA-MB-231) through endocytosis mediated *via* the HA-CD44 receptor. As a new targeting ligand, PBA can bind to the overexpressed sialic acid receptor and is often used to modify the surface of nanocarriers. Zhou et al. 35 designed a PBA-modified prodrug micelle (PBA-PEG-SS-PCL-*hyd*-DOX) with a GSH/acid response performance (Figure 2B). PBA increased internalization of the prodrug micelles by HepG2 cells and the cytotoxicity of DOX. In addition, Tang et al. 51 prepared PBA-modified magnetic mesoporous silicon nanoparticles. *In vivo* and* in vitro* experimental results confirmed that an external magnetic field and PBA could not only increase enrichment of DOX-loaded nanoparticles in tumor tissues but also increase the amount of DOX in HepG2 cells. Thus, compared with other control groups, external magnetic field and PBA achieved better antitumor effects of PBA-modified magnetic mesoporous silicon nanoparticles.

### Promoting tissue penetration

NDDSs can not only improve the solubility of chemotherapeutics but can also reduce the toxicity of systemic chemotherapy to normal tissues. Currently, commonly used chemotherapy drugs include DOX, cisplatin, paclitaxel (PTX), and camptothecin (CPT). At present, chemotherapeutic drugs are mainly loaded in NDDSs in two forms: physical loading and chemical grafting. For example, Kinoh et al. 52 linked the epirubicin (Epi) to polyaspartic acid through a pH-sensitive hydrazide bond, and then loaded staurosporine *via* intermolecular interactions to obtain dual-drug-loaded copolymer micelles. Although nanomedicines have often shown greatly enhanced therapeutic efficacy in preclinical studies compared with traditional small molecule drugs, their efficacy in the clinical setting is suboptimal due to the heterogeneity of the EPR effect and the biological barriers of tumors hindering effective penetration of NPs 53. It has been found that the extracellular matrix (ECM) 54 and a high interstitial fluid pressure (IFP) 55 in tumor tissues form a primary biological barrier to prevent nanomedicines from penetrating into tumor tissues, which is the reason why we frequently observe that nanomedicines are located around the tumor blood vessel walls of tumors and rarely diffuse deeper into tumor tissues 56. Tumor penetration of nanomedicines is highly dependent on their physicochemical characteristics (size, surface charge, and particle shape), therefore, it is very helpful to explore effective strategies to enhance therapeutic tumor penetration by tuning these factors.

#### Switchable size

**Figure 3:**
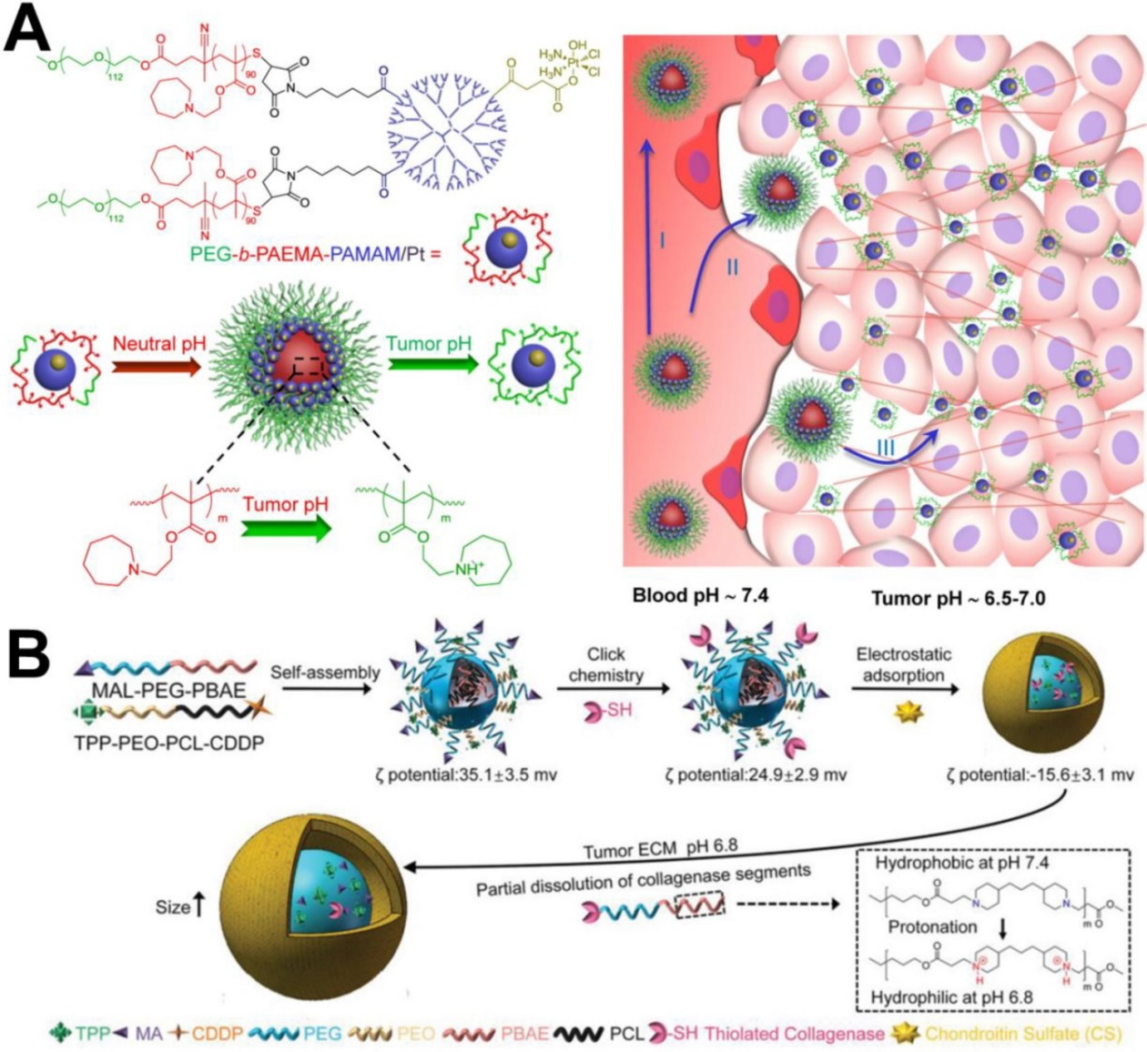
(A) Schematic illustration of the self-assembly of PEG-*b*-PAEMA-PAMAM/Pt into the pH-sensitive cluster nanobombs (SCNs/Pt) at neutral pH and the disintegration of SCNs/Pt into small particles at tumor acidic pH 59. Copyright 2016, ACS Publications. (B) Fabrication and response of size-changeable collagenase-modified nanoscavenger (CS/Col-TCPPB NPs) 66. Copyright 2020, Wiley-VCH.

It has been reported that particles with small diameter show higher tumor penetration efficiency than large particles 57, but with a smaller particle size, less retention of particles in tumor tissue occurs because they are able to re-enter the bloodstream or are filtered by the renal system. Therefore, to solve this problem, it may be an efficient strategy to design nanocarriers with changeable particle size to achieve enhanced tumor penetration. For example, Wang et al. 58 designed and synthesized the polymer prodrug PCL-CDM-PAMAM/Pt conjugated with cisplatin and the amphiphilic copolymer PEG-*b*-PCL. The two polymer chains formed micelles *via* self-assembly. At physiological pH, the micelles hold a size of approximately 100 nm and have a high propensity for long blood circulation and enhanced tumor accumulation through the EPR effect. An acidic environment (pH 6.8) triggers the release of small dendrimers of polyamide-amine (PAMAM) prodrugs that enable deep and uniform tumor penetration to reach more cancer cells. Similarly, this research group 59 also prepared PEG-*b*-PAEMA-PAMAM/Pt nanoparticles with the ability to penetrate tumor tissue. The difference was that the polyethyl methacrylate (PAEMA) in the nanoparticle structure was hydrophobic under physiological conditions and could become hydrophilic under acidic conditions. Therefore, the size of the nanoparticles was approximately 80 nm in the blood circulation and decreased after arriving at the tumor microenvironment because PAEMA changed from hydrophilic to hydrophobic, which was conducive to tissue penetration (Figure 3A).

#### Switchable surface charge

Surface charge is another influential physicochemical characteristic of nanomedicines due to their penetration into tumors. Some studies have shown that cationic nanoparticles target tumor endothelial cells and exhibit higher vascular permeability than neutral or anionic nanoparticles 53. However, cationic nanoparticles easily adhere to the tumor ECM, decreasing their effective diffusivity 60. To prolong blood circulation and promote efficient tumor uptake/penetration, nanoparticles (NPs) that are neutral or have a negative surface charge are preferred. Once NPs reach tumor tissue, positive charges are necessary to enhance tumor retention and cellular internalization through strong electrostatic interactions with negatively charged cell membranes. Therefore, designing nanocarriers with a switchable surface charge is an important way to address the above question.

Chen et al. 61 reported a pH-responsive zwitterionic poly(carboxybetaine) (PCB)-like zwitterion-modified nanomedicine with zwitterionic-to-cationic (ZTC) charge conversion ability (denoted as ZTC-NMs) for CPT delivery. ZTC-NMs showed high stability during blood circulation due to their nanosized diameter and PCB-like zwitterionic surface modification. After entering tumor tissue, the amide bond formed between 2,3-dimethylmaleic anhydride (DMMA) and the amino group responded to the acidic tumor microenvironment and achieved acid-responsive cleavage, leading to ZTC surface charge conversion of the ZTC-NMs. Then, the highly positive quaternary ammonium salt could induce rapid internalization of the NPs by tumor cells through effective electrostatic interactions with negatively charged cell membranes. Therefore, the ZTC charge conversion property can improve the cellular internalization efficiency of NMs and thus promote efficient drug penetration.

#### Particle shape

In addition to the above strategies, morphology can also improve penetration of nanocarriers into tumor tissues and enhance the effect of chemotherapy. For example, Zeng et al. 67 designed a worm-like drug-loaded micelle (RNW) with tumor targeting and pH responsiveness. The drug-loaded micelles could not only actively target tumor cells, but also had strong tumor penetration and on-demand drug release capability. The system was formed from a pH-responsive amphiphilic copolymer of methoxypoly (ethylene glycol)-block-poly(2-diisopropyl methacrylate) (mPEG-*b*-PDPA), and disulfide-linked RGD-targeted cytotoxic drug (DM1) conjugates (RGD-SS-DM1). Drug-loaded micelles have the following advantages: 1) they can accurately target brain tumors due to their worm-like structure with the ability to pass through the blood-brain barrier; 2) they have better tumor penetration and internalization efficiency by tumor cells; and 3) they respond to the tumor microenvironment (acidic and reducing substances) and then release drugs as needed. The results of* in vivo* experiments confirmed that this system had a good inhibitory effect in an in-situ glioma model with an inhibition rate of 88.9%.

#### Scrapable extracellular matrix

The extracellular matrix (ECM) (*e.g.,* HA and collagen fibre) has become one of the most important factors that can seriously prevent deep penetration of NPs in the intercellular space. Therefore, researchers have been studying how to degrade the extracellular matrix and increase the penetration of NPs. Certain exogenous enzymes can consume tumor matrix components and can be used to improve the penetration of NPs. For example, Zhou et al. 62 designed HPEG-PH20-NPs nanocarriers containing human hyaluronidase PH20 (rHuPH20). It confirmed that the HPEG-PH20-NPs had a good ability to remove hyaluronic acid *in vitro*. In addition, *in vivo* antitumor experiments confirmed that DOX-loaded HPEG-PH20-NPs could better inhibit the growth of 4T1 breast cancer tumors. In another report, Liu et al. 63 used hyaluronidase to degrade hyaluronic acid in the tumor microenvironment, which improved tissue permeability to improve drug diffusion. Recombinant long-acting hyaluronidase was constructed through genetic engineering technology to improve the bioavailability of subcutaneously administered of macromolecules, and to increase the therapeutic effect of anticancer drugs *in vivo*. It was verified that collagenase could break down the ECM and enhance the interstitial diffusion rate of nanocarriers in tumor tissue 64. For example, Dong et al. 65 proposed a strategy that employed nitric oxide (NO) to activate endogenous matrix metalloproteinases (MMP-1 and MMP-2) and to induce collagen consumption to improve drug penetration in solid tumors. In their report, mesoporous silica nanoparticles (MSNs) were used as a NDDSs to load DOX and NO donors (*S*-nitrosothiol) simultaneously, obtaining DN@MSNs. NO-loaded MSNs could induce MMP activation, which led to collagen degradaion in the tumor extracellular matrix, thereby enhancing the penetration of both the nanovehicle and DOX into the tumor tissue and significantly improving the antitumor effect of chemotherapy with no obvious systemic side effects. Xu et al. 66 developed novel size-changeable collagenase-modified polymer micelles (named CS/Col-TCPPB) to simultaneously enhance penetration and retention of nanocarriers in deep tumor tissue to enhance cancer therapeutic efficiency. The preparation process can be found in Figure 3B. After CS/Col-TCPPB arrived at the tumor site, the PBAE segments turned from hydrophobic to hydrophilic due to protonation of the tertiary amino group in the acidic tumor environment (pH 6.8), thereby exposing collagenase, promoting enzymatic digestion of collagen fibres, and enhancing the intratumoral penetration of the drug-loaded nanoparticles.

## Chemotherapy towards specific subcellular organelles

To achieve the therapeutic effects of chemotherapeutic drugs according to their respective mechanisms of action, it is well known that almost all of them must be targeted to specific sites of action 68. For example, DOX can induce cancer cell apoptosis by inhibiting the activity of topoisomerase Ⅱ and damaging DNA, while PTX can cause cell death by inhibiting the microtubule depolymerization in the cytoplasm 69. Therefore, to obtain a satisfactory therapeutic outcome, the ideal approach is to ensure that the appropriate therapeutic agents with optimal concentration can be located at the right place. For these reasons, simply delivering therapeutic agents into tumor tissues or cells is not sufficient enough to obtain the desired therapeutic effect. To tackle this problem, it is particularly important to achieve targeted delivery of therapeutic agents to subcellular organelles, which is the best strategy to completely eradicate tumors and prevent tumor recurrence, invasion, and metastasis 70, 71. At present, research on targeted subcellular organelle delivery based on NDDSs mainly includes targeting mitochondria and nuclei.

### Mitochondria-targeted chemotherapy

**Figure 4:**
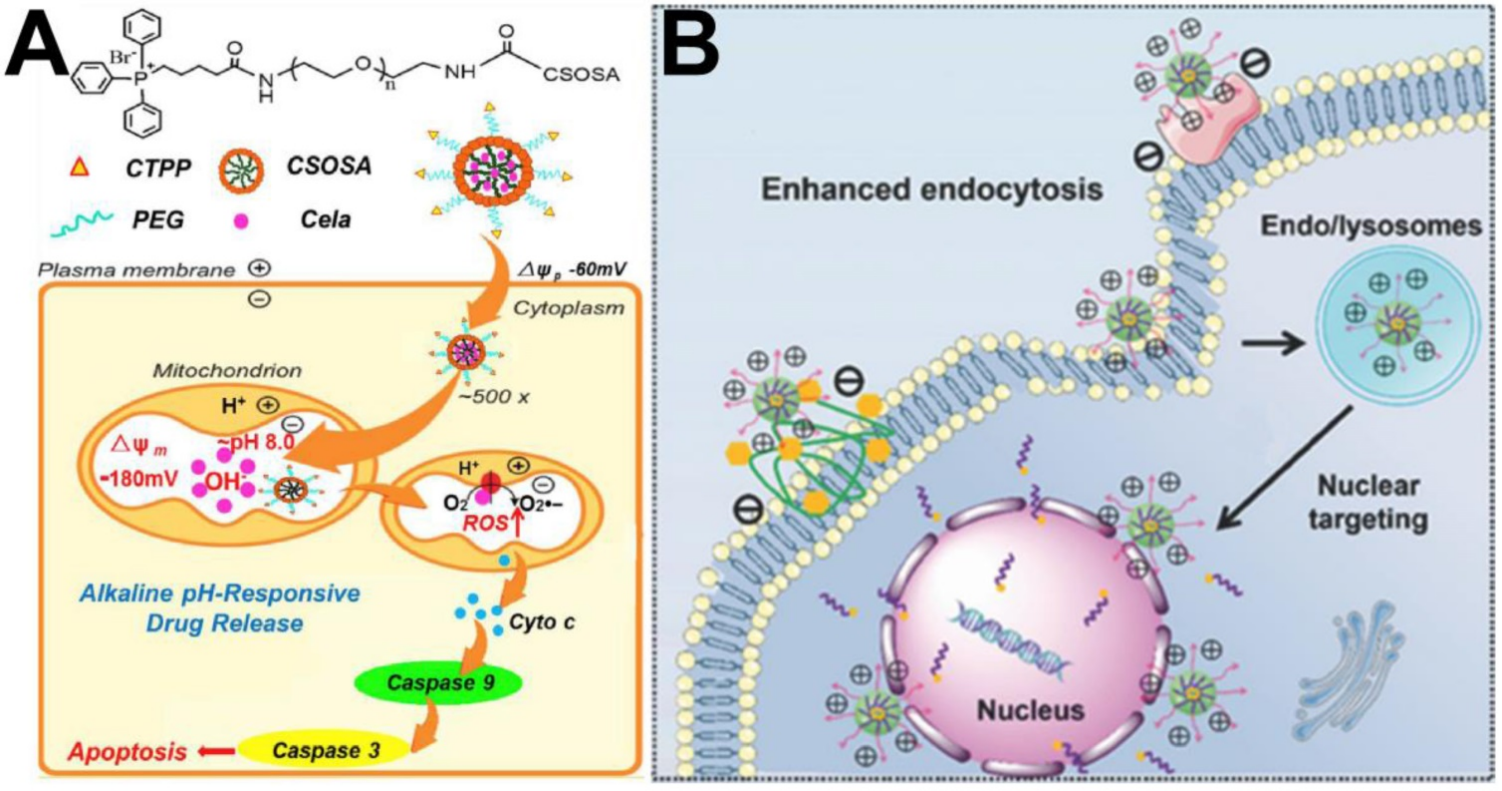
(A) The schematic illustration of drug delivery system with mitochondrial alkaline pH-responsive release 74. Copyright 2018, Elsevier. (B) The schematic illustration of Tat-mediated enhanced endocytosis into tumor cells and nuclear targeting 77. Copyright 2018, Wiley-VCH.

In the past several decades, anticancer strategies based on targeting mitochondria have received much attention due to their crucial functions in the cell. To date, triphenylphosphine (TPP) is the most commonly used functional group for transporting mitochondriotoxic agents to mitochondria because it can embed into the mitochondrial membrane 72. Yu et al. 73 constructed a pillar arene-based rotaxane (R1) by using tetraphenylethene (TPE) and TPP moieties as stoppers; the TPE unit acted as the aggregation-induced emission (AIE) reagent, and the TPP group was used as a mitochondria-targeted unit. DOX was introduced into R1 through acid-sensitive bonds to form fluorescence resonance transfer (FRET)-capable DOX-loaded nanoparticles.* In vitro* cell experiments confirmed that large amounts of DOX could be released from the DOX-loaded nanoparticles and enriched in mitochondria to kill cancer cells after nanoparticles are internalized by HeLa cells. In another report, Tan et al. 74 produced mitochondria-targeted nanocarriers (CTPP-CSOSA) by choosing the lipophilic cation (4-carboxybutyl) triphenylphosphonium bromide (a type of TPP cation, CTPP) to modify glucolipid-like conjugates (CSOSA). Celastrol-loaded micelles (CTPP-CSOSA/Cela) selectively targeted mitochondria and responded to the mitochondrial alkaline pH environment (pH 8.0) and released Cela, which induced reactive oxygen species (ROS) generation, further activating a cascade of Caspase 9 and Caspase 3 reactions and promoting tumor cell apoptosis by regulating mitochondrial signalling pathways (Figure 4A).

### Nucleus-targeted chemotherapy

Because the final destination of many first-line chemotherapeutics (*e.g.,* DOX, cisplatin, and CPT) is DNA or its associated enzymes in the nucleus, these drugs must be transported into cellular nuclei to exert their anticancer effect 70. Unfortunately, an agent transported from the cytoplasm into the nucleus must pass through the nuclear pore complexes (NPCs) 75, and only sufficiently small molecules can enter nuclei through passive diffusion, especially in proliferating cells. Therefore, the development of nuclear-targeted drug delivery mostly relies on nanocarriers with nuclear accumulation capacity. At present, the design ideas for nuclear-targeted nanocarriers can be summarized into the following two types: 1) modification of nanocarriers with nuclear targeting peptides to facilitate nuclear enrichment and 2) preparation of nanocarriers with large-to-small size-changeable performance after internalization by cancer cells to activate nuclear entry.

The positively charged TAT peptide has been demonstrated to promote nuclear delivery of TAT-modified nanoparticles, which significantly enhances nuclear drug delivery 76. For example, Zhou et al. 77 designed multifunctional micelles with high nuclear targeting of therapeutics, which were constructed from poly(ethylene glycol)-poly(ε-caprolactone) with 2,3-dimethylmaleic anhydride-TAT decoration (PECL/DA-TAT). As shown in Figure 4B, in a mildly acidic environment (pH 6.8), these micelles facilitated cell internalization and subsequent nuclear targeting of the chemotherapeutic 10-hydroxycamptothecin, and obviously enhanced cytotoxicity against 4T1 and A549 cells.

In addition, as confirmed in previous studies, small-sized NPs (<50 nm) have the advantage of passing through NPCs and efficient nuclear uptake. Therefore, depending on the specific microenvironment in the cancer cell, size-changeable nanocarriers are a promising drug delivery system to actively transport chemotherapeutic drugs to cancer cell nuclei. That is, the size of the nanoparticles at the initial stage should be large enough to reduce renal clearance and maintain a good EPR effect, but once internalized by the cancer cell, the NPs are able to decrease to a smaller size for nuclear uptake. For example, in a “proof-of-concept” study, Zhou et al. 78 designed mPEG-PLA-ss-PEI-DMMA (PELEss-DA) polymer micelles as drug delivery systems with variable sizes from large into small to facilitate nuclear entry and release of therapeutics in the nucleoplasm. In this well-defined core-corona structure, a polylactide (PLA) segment was used as the core, and two water-soluble polymers, namely methoxy poly(ethyleneglycol) (mPEG) and polyethyleneimine (PEI), were used as the corona material. Specifically, the positive charges of PEI were masked through amidation to ensure good stability and long blood circulation of the carriers under physiological conditions (pH 7.4). Due to the charge reversal and subsequent size enlargement in acidic pH tumor tissues, greater cell internalization and faster lysosome escape *via* the proton sponge effect of PEI occurred. Then, due to deshielding of the PEI shell *via* the cleavage of disulfide bonds by intracellular GSH, a sufficiently small PELEss-DA micelle was produced, which could effectively transport the drug into the cell nucleus.

In addition, external stimuli-triggers (*e.g.,* light and ultrasound) have also been used to change the size of nanocarriers to achieve drug delivery into the nucleus. For example, Tan et al. 79 developed a size-photocontrollable nanoplatform *via* DNA hybridization, in which a small nucleus-uptake nanodrug system (DOX-loaded gold NPs) was assembled onto a larger cell-targeted near-infrared light (NIR)-responsive silver-gold nanorod (NR). *In vitro* experimental results showed that the photothermal effect of the NR under NIR irradiation caused DNA dehybridization and release of the NPs, which further entered the nuclei using the advantage of their small particle size. Therefore, this nanoplatform promoted accumulation of the anticancer drug DOX at its target site.

### Others

Because endo/lysosome, the golgi apparatus, endoplasmic reticulum (ER), and other subcellular organelles have certain roles in cancer cells, they can also be studied as chemotherapy targets. For example, Gong et al. 80 developed a Golgi apparatus-targeting prodrug nanoparticle system by synthesizing retinoic acid (RA)-conjugated chondroitin sulfate (CS) (CS-RA). The prodrug nanoparticles appeared to accumulate in the Golgi apparatus in cancer cells and improve RA release in an acidic environment. In addition, DOX and cisplatin can be directly transported into the ER, lead to cancer cell death *via* severe ER stress. Similarly, PTX can induce lysosomal membrane permeabilization (LMP) and activate the lysosomal cell death pathway in cancerous cell, therefore it can be transported into lysosomes to kill cancer cells. In our opinion, this study area will open a new paradigm for precise and high-performance cancer therapy by exploring new subcellular-targeted chemotherapies.

## Reversing resistance mechanisms

MDR encompasses a broad spectrum of defence mechanisms by cancer cells, which makes them resistant to one or more chemotherapeutic drugs by decreasing uptake, increasing efflux, inactivating drugs, activating DNA repair mechanisms, upregulating metabolism, and/or stimulating detoxification pathways 81. Therefore, the failure of chemotherapy against cancer often occurs because of MDR 82, 83. To overcome MDR, the traditional approach is using higher doses or greater frequencies of chemotherapeutic agents 84. Although these measures can improve antitumor efficacy to a certain extent, they are far from optimal due to the serious side effects or toxicity to healthy tissues and organs caused by nonspecific treatments. Hence, there is a strong incentive to develop other optimized strategies to overcome MDR to maximize the therapeutic index of anticancer drugs.

At present, research has focused on investigating the molecular pathways mediating MDR in an effort to develop rational strategies for intervention. Overexpression of the ATP-binding cassette (ABC) is the major molecular mechanism of MDR. In particular, P-glycoprotein (P-gp) is one of the main ABC transporter proteins and is overexpressed on the cell-membrane. P-gp has the ability to pump cytotoxic agents out of cells 84. Therefore, overcoming the P-gp-mediated MDR mechanism is one of most widely employed strategies to improve the effect of chemotherapeutics on MDR tumors 85. Although NPs can enhance chemotherapeutic uptake by cells *via* phagocytosis, which can bypass the efflux action of P-gp, and active targeting ligands on the surface of nanoparticles can also facilitate the evasion of the P-gp pathway* via* receptor-mediated endocytosis 84, these measures cannot fundamentally solve the problem of MDR. Consequently, combination chemotherapy with P-gp inhibitors is considered the most reliable strategy and is applied for treatment of multidrug resistant tumors.

**Figure 5:**
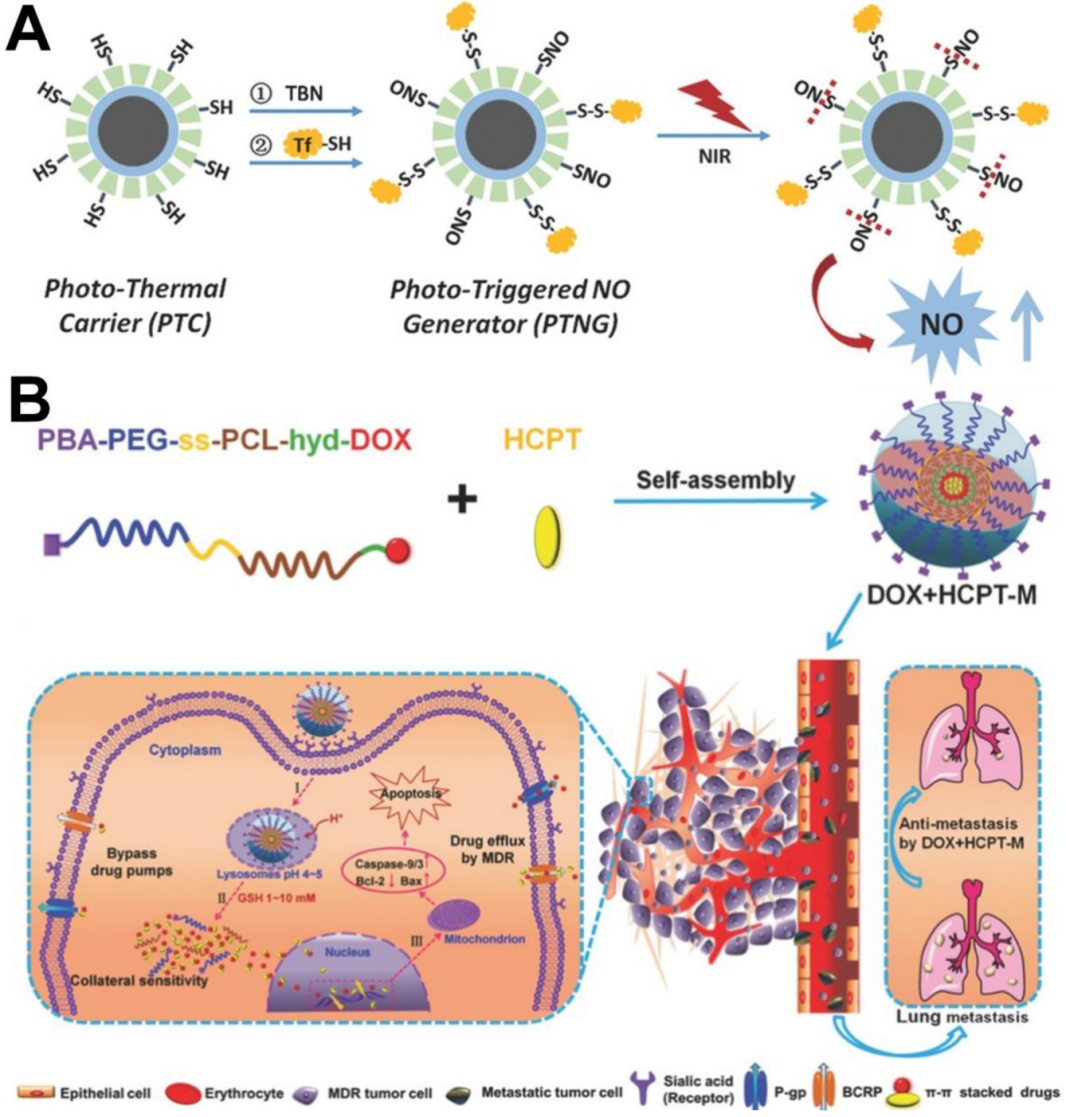
(A) Schematic illustration of the fabrication of phototriggered NO nanogenerators (PTNGs) 97. Copyright 2017, Wiley-VCH. (B) Schematic illustration of combination of DOX and HCPT using a multifunctional micelle to combat MDR and lung metastases 98. Copyright 2016, Wiley-VCH.

P-gp inhibitors can be classified into organic small molecules (*e.g.* cyclosporin A 86, vitamin E 87, verapamil hydrochloride 88 and curcumin 89), small interfering RNA (siRNA) 90, gas molecules (H_2_ 92 and NO 93) and ions (*e.g*. Ca^2+^ 91). Among them, siRNA and gas molecules are widely used to overcome MDR. For example, Cheng et al. 94 successfully developed a multifunctional nanoplatform (M-R@D-PDA-PEG-FA-D) that integrates chemotherapy/photothermal therapy/gene therapy. Because the nanocarrier could simultaneously deliver siRNA and DOX into MCF-7/ADR cells, *in vitro* western blotting results showed that the expression of P-gp protein could be effectively downregulated by siRNA, resulting in higher concentrations of DOX in the MCF-7/ADR cancer cells to significantly improve chemotherapy. In addition, because NO can inhibit expression of the P-gp, it is also often used as a P-gp inhibitor to reverse MDR tumors 95. For example, Chen et al. 96 designed a nanomicelle mPEG-PLGA containing both BNN6 and DOX; BNN6 could decompose and produce NO to inhibit DOX efflux mediated by P-gp under light conditions. In another report, Yang et al. 97 designed NPs that could reverse MDR by inhibiting overexpression of P-gp *via* NO. The difference in this design was that the NO donor (nitrosothiol, SNO) was connected to the surface of the nanoparticle through a chemical bond and could be decomposed to produce NO *via* heat (Figure 5A).

In addition to using P-gp inhibitors, Zhou et al. 98 developed another new strategy to evade drug pumps recognition by co-delivering *π-π* stacked dual anticancer drugs (DOX and CPT). As shown in Figure 5B, the DOX prodrug copolymer PBA-PEG-ss-PCL-hyd-DOX, which was synthesized by conjugating DOX to the hydrophobic chain of the polymer backbone *via* a pH-responsive hydrazine bond, could encapsulate HCPT *via π-π* stacking between DOX and HCPT to obtain dual drug-loaded micelles named DOX+HCPT-M. The results of *in vitro* experiments verified that the released drugs with complex aromatic *π-π* conjugated structures could evade recognition by drug pumps due to a slight change in the molecular structure of the drugs and display high therapeutic efficacy in MCF-7/ADR cancer cells.

## Combined chemotherapy

A single therapeutic modality is one reason for the undesirable outcomes of clinical chemotherapy, and it is difficult to achieve satisfactory anticancer effects. Compared to monochemotherapy, combination regimens can target different therapeutic pathways in cancer cells and lower drug doses to reduce side effects. Therefore, in recent years, combination approaches for enhancing cancer chemotherapy have been explored, such as chemotherapy combined with photodynamic therapy, photothermal therapy, chemodynamic therapy, radiotherapy, gas therapy, and immunotherapy and multiple therapy approaches (see Table 2).

### Combination with photodynamic therapy (PDT)

Chemotherapy combined with PDT is one of the most common strategies to improve anticancer effects. Because PDT functions as a cancer treatment strategy and is associated with cytotoxic ROS, which are produced by photosensitizers (*e.g.,* porphyrin, chlorine e6, indocyanine green, and Rose Bengal) under specific light irradiation 99, 100, coadministration of photosensitizers and chemotherapeutic drugs is involved. For example, Park et al. 101 designed a highly tumor-specific light- triggered drug delivery system (H-LTDC) composed of chondroitin sulfate (CS) and pheophorbide-a (photosensitizer) joined through covalent bonding. Under 670 nm laser irradiation, DOX-loaded H-LTDC could generate ROS to induce degradation of CS, leading to DOX release for chemotherapy. Furthermore, the generated ROS could also be used to kill cancer cells. In particular, although the hypoxic environment of tumor tissue affects PDT efficacy, it is good for several hypoxia-activated prodrugs. For example, tirapazamine (TPZ) exhibits highly selective cytotoxicity toward hypoxic cancer cells. Therefore, combining PDT and TPZ-mediated hypoxia-activated chemotherapy could be promising for enhanced anticancer therapy 102. To date, researchers have used the characteristics of nanomaterials to design different nanomedicines containing TPZ. For example, Wang et al. 103 developed iRGD-modified nanoparticles for simultaneous tumor delivery of the photosensitizers indocyanine green (ICG) and TPZ. *In vitro* and *in vivo* experimental results showed that the nanoparticles could significantly improve penetration in both 3D tumor spheroids and orthotopic breast tumors. In addition, under NIR irradiation, ICG-mediated photodynamic therapy induced oxygen consumption and aggravated the hypoxic environment of cancer cells, which further activated the anticancer activity of the codelivered TPZ for a synergistic cell-killing effect.

**Figure 6:**
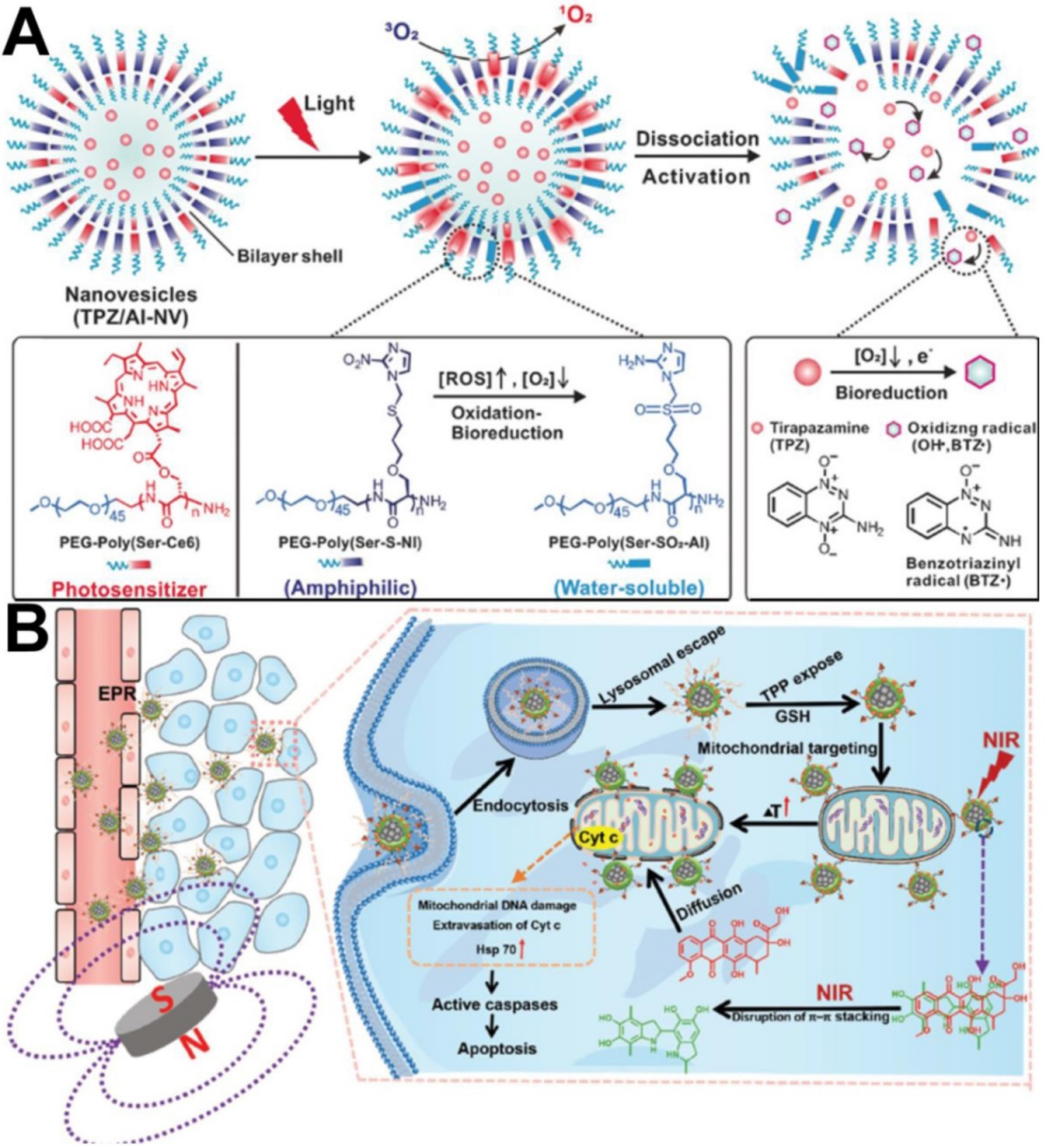
(A) Formation and mechanism of TPZ/AI-NV for dissociation of vehicles and simultaneous activation of bioreductive prodrug 104. Copyright 2017, Wiley-VCH. (B) Schematic of F@PDA-TPP/SS/DOX for synergism of PTT and chemotherapy 106. Copyright 2018, Wiley-VCH.

Subsequently, Qian et al. 104 designed a polymeric nanovesicle (TPZ/AI-NV), which was assembled by combining the photosensitizer chlorine e6 (Ce6)-modified diblock copolymer PEG-Poly(Ser-Ce6) and 2-nitroimidazole (NI) with the thioether-modified diblock copolymer PEG-Poly(Ser-S-NI), to enhance the hypoxia-activatable chemotherapy, as shown in Figure 6A. Compared to the above design, the difference is that the photosensitizer was Ce6, and it was grafted with the main chain of the polymer through a covalent bond, which greatly increased the drug loading capacity and prevented early release of Ce6. In addition, to better release TPZ in cancer cells, the hypoxia-sensitive 2-nitroimidazole (NI) structure was introduced into the backbone. *In vitro* and *in vivo* experiments indicated that this nanovesicle efficiently induced apoptotic cell death and significantly inhibited tumor growth.

### Combination with photothermal therapy (PTT)

Chemotherapy combined with PTT is another common combination therapy strategy and shows great potential to optimize cancer therapy. PTT functions as a physical treatment modality because it is associated with hyperthermia induced by laser irradiation, which increases the local temperature to cause cellular damage in tumors. Various photothermal agents can induce hyperthermia, such as metal-based nanomaterials, carbon-based nanomaterials, magnetic nanoparticles, and organic dyes. When co-administrated with chemotherapeutic agents, these photothermal agents can enhance chemotherapeutic outcomes because hyperthermia can increase vascular permeability within tumors to promote intratumoral transport of drugs 5. For example, Su et al. 105 designed a novel porphyrin-based micelle, which was formed *via* self-assembly of a hybrid amphiphilic polymer mPEG-PLGA-porphyrin, and the micelle could be used to load DOX and TAX simultaneously* via* an improved double-emulsion method. Under NIR irradiation, the dual-drug-loaded micelles could generate high heat and release dual drugs to co-kill the cancer cells. More importantly, the combined strategy of PTT and chemotherapy conferred great chemosensitivity to cancer cells and achieved tumor regression using approximately 1/10 of the traditional drug dosage.

In addition to the use of photothermal agents, nanocarriers themselves also have photothermal effects, which can be used for delivering chemotherapeutic drugs to achieve synergism between PTT and chemotherapy. For example, Wang et al. 106 reported a multistage targeting strategy using magnetic composite nanoparticles to provide synergistic PTT and chemotherapy. The magnetic composite nanoparticles (named F@PDA-TPP/SS) were composed of four units: Fe_3_O_4_ colloidal nanocrystal clusters (Fe_3_O_4_ CNCs) as a core, a polydopamine (PDA) inner shell as the PTT agent functionalized with triphenylphosphonium (TPP) for mitochondrial targeting, and the mPEG outer shell linked by disulfide bonds (ss). DOX was loaded in F@PDA-TPP/SS *via π-π* stacking between the aromatic regions of PDA and DOX. Under NIR irradiation, the nanoparticles not only rapidly produced a large amount of heat for PTT, but also triggered rapid drug release for chemotherapy at the same time (Figure 6B).

### Combination with chemodynamic therapy (CDT)

In recent years, CDT has become an interesting research topic due to its advantages of higher tumor specificity and selectivity, low systemic toxicity, and few side effects. Most importantly, compared to PDT/PTT, CDT does not require a specific stimulation in the treatment process 107. Furthermore, the combination of chemotherapy and CDT can not only reduce the side effects of chemotherapeutic drugs, but can also enhance CDT efficacy. For example, Xue et al. 108 reported a DOX-loaded and modified HA metal-organic framework (MOF) material (named MIL-100@DOX-HA, DMH) for combined CDT and chemotherapy against cancer. Because of the high DOX loading efficiency, DMH could achieve better chemotherapy effects and could produce a large amount of toxic •OH for CDT through Fenton reactions. Therefore, DMH NPs have immense potential for reducing systemic toxicity and improving the therapeutic effect of DOX against breast cancer.

**Figure 7:**
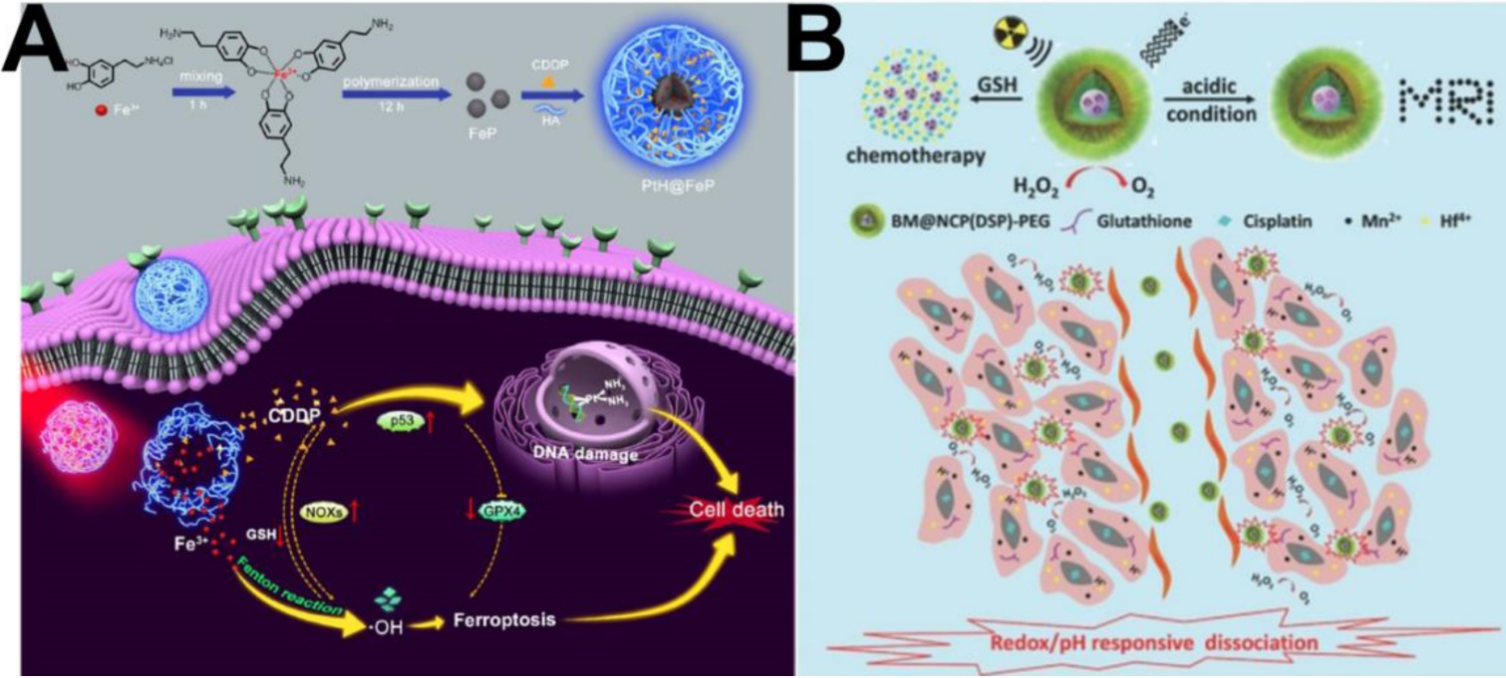
(A) Schematic illustration of PtH@FeP-mediated antitumor synergistic therapy by combining CDDP-induced apoptosis and CDT-based ferroptosis 113. Copyright 2020, ACS Publications. (B) Scheme illustrating the redox/pH responsive behaviors of BM@NCP(DSP)-PEG composite nanoparticles in the tumor microenvironment for cancer chemoradiotherapy 114. Copyright 2017, Wiley-VCH.

Similarly, due to its good killing effect on cancer cells, the cell death pathway iron-based ferroptosis has received widespread attention in recent years 109. To the best of our knowledge, Fenton/Fenton-like reactions have been illuminated as a clear mechanism to induce ferroptosis in tumor cells through ROS upregulating 110, 111. In particular, the activated cisplatin can specifically elevate the intracellular H_2_O_2_ level through cascade reactions. Therefore, cisplatin prodrug-based chemotherapy is an ideal treatment partner for CDT because of its ability to supply H_2_O_2_ for the Fenton reaction, thereby obtaining synergetic CDT-chemotherapy 112. For example, Yu et al. 113 successfully prepared a core-shell platform from HA-cisplatin (PtH) cross-linked complexes placed onto a Fe(III)-polydopamine (FeP) core to obtain PtH@FeP for combined CDT-chemotherapy. As shown in Figure 7A, suppression of GPX4 and activation of NOXs subsequently resulted in a high level of H_2_O_2_ and lipid peroxidation owing to the participation of CDDP, indirectly resulting in the ferroptosis effect. In addition, the FeP core endowed the nanocarriers with CDT capability, which not only exerted direct tumor elimination activity but also benefited ferroptosis and CDDP cytotoxicity. This work developed a promising strategy for designing ferroptosis-assisted multidrug chemotherapy by amplifying intratumoral oxidative stress. In short, the combination of CDT and chemotherapy can realize a satisfactory treatment performance through a significant synergistic effect and thus has broad potential applications.

### Combination with radiotherapy

Radiotherapy is one of the most widely used and effective approaches for cancer treatment in clinic, therefore, the combination of chemotherapy and radiotherapy has also been reported. For example, Liu et al. 114 used the coordination principle of metal ions and organic molecules to synthesize a nanometal complex carrier named BM@NCP (DSP)-PEG containing both a cisplatin prodrug and the radioactive element Hf. The agent Hf ion and the cisplatin prodrug were attached to the surface of bovine serum albumin (BSA) stabilized manganese dioxide (MnO_2_) nanoparticles *via* metal coordination, and then, the nanoparticles were modified with PEG to increase their biocompatibility. As shown in Figure 7B, the MnO_2_ core could catalyze *in situ* generation of O_2_ from decomposition of the endogenous H_2_O_2_ produced in tumor cells, to overcome the hypoxic environment of tumor tissues and enhance the effect of radiotherapy. In addition, the cisplatin prodrug was cleaved under the condition of a high GSH concentration and then produced a chemotherapeutic effect. The acidic environment also promoted the production of Mn^2+^ for imaging effects. Therefore, this system contains three functions: imaging functions based on radiotherapy and chemotherapy.

### Combination with gas therapy

**Figure 8:**
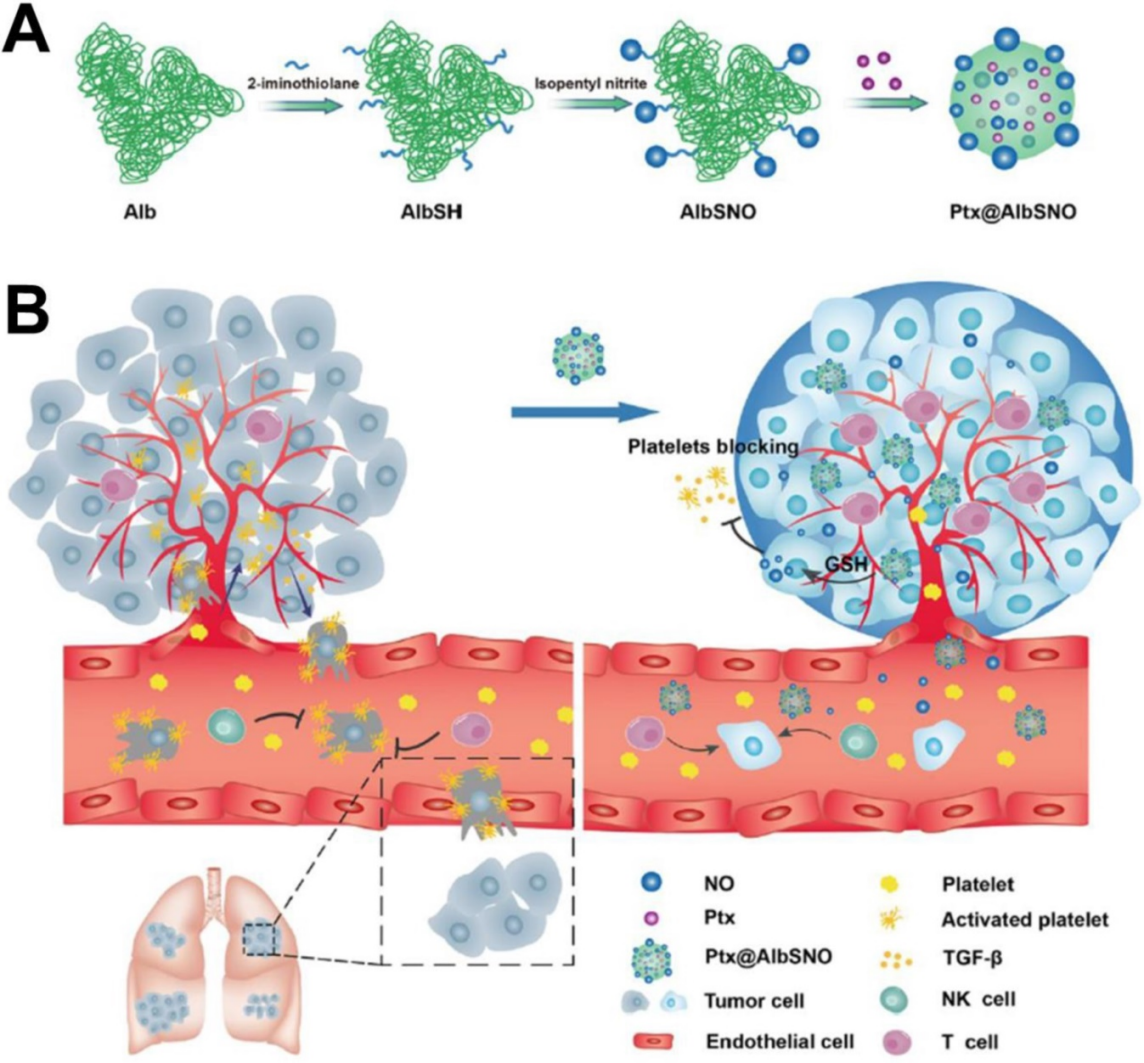
(A) Schematic of AlbSNO and Ptx@AlbSNO preparation. (B) Tumor-specific release of NO from Ptx@AlbSNO possesses the dual functions of simultaneously preventing tumor metastasis and reversing tumor immunosuppression by blocking platelets 116. Copyright 2020, ACS Publications.

Among new therapies, gas therapy (*e.g.,* NO, hydrogen sulfide, and carbon monoxide) has been developed as an emerging “green” approach for cancer therapy because it does not induce drug resistance and has minimal side effects in normal tissues 115. However, gas is a concentration-dependent “double-edged sword” in the body, which may limit the effect of gas treatment alone. In recent years, gas therapy-based combined cancer therapy, called gas-chemotherapy, has emerged and significantly improves antitumor efficiency by generating synergistic effects. For example, Li et al. 116 designed a tumor microenvironment-responsive NO release nanoparticle (named Ptx@AlbSNO) that was able to specifically and safely codeliver a NO donor and PTX into tumor tissues. As shown in Figure 8, the Ptx@AlbSNO NPs could respond to GSH in the tumor cell and specifically release NO and PTX for synergistic cytotoxicity to suppress primary tumor growth. Furthermore, the released NO inhibits platelet aggregation and adhesion to prevent tumor cell epithelial-mesenchymal transition (EMT), thereby preventing platelet adhesion around circulating tumor cells (CTCs) and reducing distant metastasis. Therefore, NO-based nanoplatforms provide a simple strategy to improve cancer treatment effects.

### Combination with gene therapy

Gene therapy, such as plasmid DNA (pDNA), siRNA, and microRNA (miRNA), has been introduced in combination with chemotherapy because it could downregulate/replace disordered genes or silence unwanted gene expression in tumor 117. For example, Kang et al. 118 explored the novel concept of engineering blood exosomes as co-delivery nanosystems, which can efficiently co-loading of DOX and nucleic acids (chol-miR21i) for cancer therapy. They demonstrated that this blood exosome-based nanosystem co-delivered DOX and chol-miR21i into tumor cells with superior tumor accumulation and improved cytosolic release, and the released drugs and RNAs simultaneously interfere with nuclear DNA activity and downregulate the expression of oncogenes, thus remarkably inhibiting the growth of tumors and alleviating side effects.

### Combination with immunotherapy

To the best of our knowledge, cancer immunity consists of five steps (called the cancer-immunity cycle) 119: 1) antigen production by cancer cells; 2) antigen delivery by antigen-presenting cells (APCs); 3) activation of T cells by antigens; 4) trafficking and infiltration of T cells to tumors; and 5) recognition and killing of tumor cells by cytotoxic T cells. Therefore, each step can be used as a potential therapeutic target to activate cancer immunotherapy. At present, the combination of chemotherapy and immunotherapy based on NDDSs is a promising approach for improving antitumor efficiency and has been widely studied in preclinical and clinical research because NDDSs can easily be internalized by immune cells and can re-educate the tumor microenvironment (TEM) due to their special physical and chemical properties, thus boosting the immune system 120, 121.

**Figure 9:**
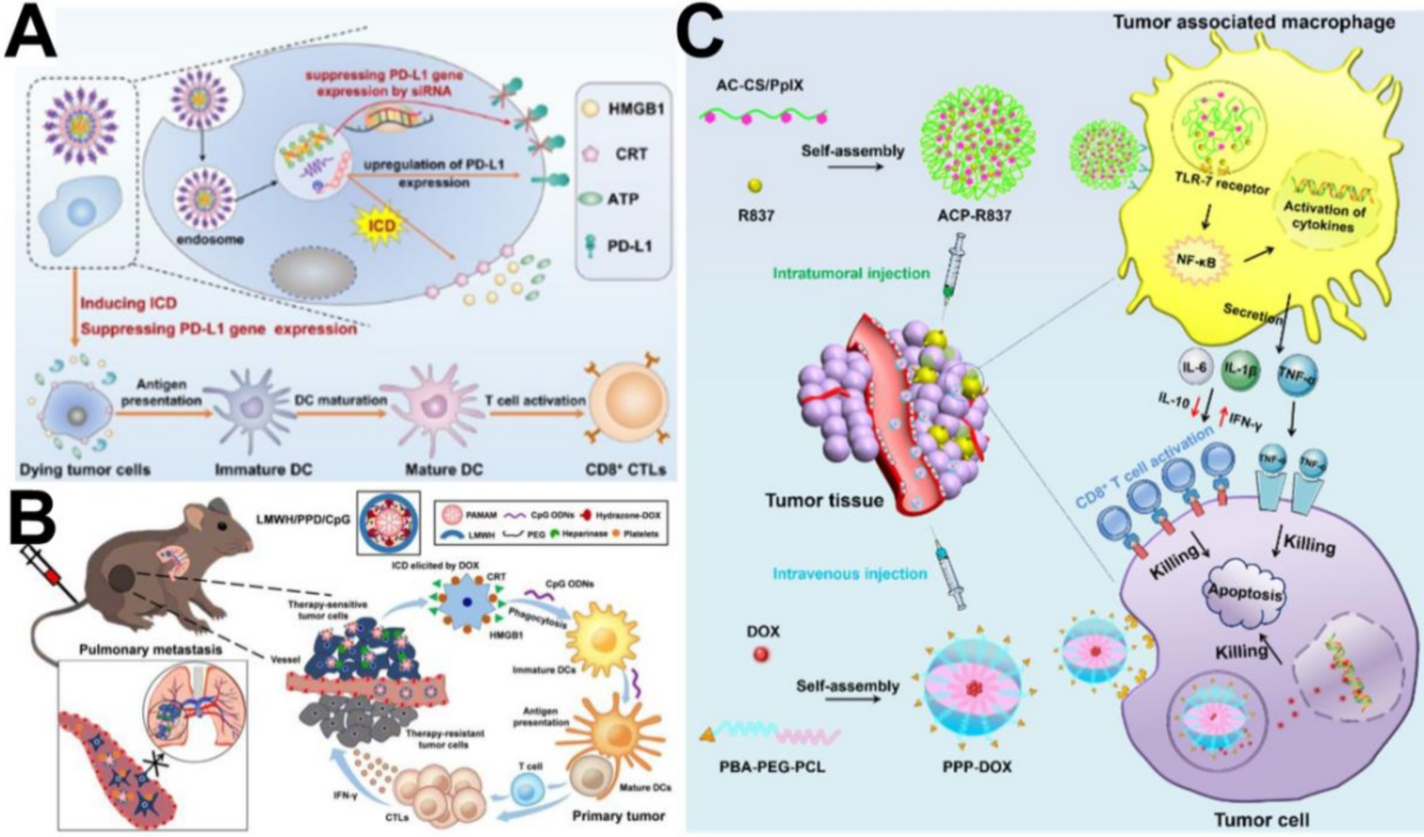
(A) Schematic illustration of the carrier-free nanoassembly PEG@D:siRNA for combinationally inducing ICD and reversing immunosuppression 124. Copyright 2020, Elsevier. (B) Schematic illustration of LMWH/PPD/CpG to inhibit melanoma primary tumor and pulmonary metastasis 127. Copyright 2019, Ivyspring International Publisher. (C) Schematic illustration of the enhanced cancer chemo-immunotherapy resulting from intratumorally and intravenously injected nanomedicines 128. Copyright 2019, Elsevier.

In recent years, immune checkpoint blockers, such as those inducing cytotoxic T-lymphocyte-associated protein 4 (CTLA-4) blockade, programmed death 1/programmed death ligand 1 (PD-1/PD-L1) blockade, and indoleamine 2,3-dioxygenase (IDO) blockade, have often been used as immunotherapeutic agents combined with chemotherapy to enhance the effectiveness of cancer treatment 122, 123. For example, Wang et al. 124 developed a facile carrier-free nanoassembly of an acid-activatable DOX prodrug and siRNA for combined induction of immunogenic cell death (ICD) and reversal of immunosuppression. As shown in Figure 9A, the carrier-free nanoassembly loaded with the two drugs was formed *via* cooperative* π-π* stacking and electrostatic interactions and termed PEG@D:siRNA. When PEG@D:siRNA was internalized by cancer cells, it released DOX and siRNA in the acidic endosomal/lysosomal compartments, and the released DOX induced ICD in tumor cells, and then provoked an antitumor immune response. At the same time, the released siRNA efficiently suppressed upregulation of the immunosuppressive gene PD-L1 during DOX-based chemotherapy, propagating antitumor immunity from ICD and enhancing cancer therapy. Similarly, Shuai et al. 125 reported pH and MMP-2 dual-sensitive polymeric micelles that could co-deliver PTX and anti-PD-1 antibody (aPD-1) for synergistic cancer chemo-immunotherapy. Both* in vitro* and *in vivo* experiments showed an enhanced antitumor effect of PTX and aPD-1 in combination chemo-immunotherapy. In another study, Yang et al. 126 reported dual pH/redox-responsive shrinkage and charge-reversal micelles that could co-deliver IDO inhibitors (NLG919) and curcumin (CUR). After the micelles were internalized by cancer cells, CUR and NLG919 were rapidly released from the micelles in the enriched GSH cellular microenvironment, leading to chemotherapy and an improved immune response for tumor therapy.

In addition, several studies have indicated that immunoadjuvants (*e.g.,* CpG, R837, and R848) can observably enhance the tumor response to chemotherapy-elicited immune responses by improving the recognition and delivery of cancer antigens by DCs. For example, He et al. 127 designed a PAMAM-based chemo-immunotherapy nanoparticle (LMWH/PPD/CpG) that could co-deliver DOX and immunoadjuvant (cytosine-phosphate-guanine oligonucleotides, CpG ONDs) for synergistic treatment of metastatic melanoma. DOX was conjugated to the amino-terminated PAMAM dendrimer by pH-sensitive hydrazine, and LMWH/PPD/CpG was added as a negatively charged anti-metastatic low molecular weight heparin (LMWH) coating on the surface of PAMAM. As shown in Figure 9B, DOX elicited tumor-specific immune responses *via* ICD, and the CpG ODNs further improved immunological effects by enhancing the maturation of DCs and then increasing the level of cytolytic T lymphocytes. R837 is also used as an immune-adjuvant to modulate the tumor-associated macrophages (TAMs) to improve tumor chemo-immunotherapy. For example, Zhou et al. 128 designed two types of targeting micelles to separately deliver DOX and R837 to tumor cells and TAMs *via* intravenous injection and intratumoral injection, respectively, for enhanced cancer chemo-immunotherapy against breast cancer. As shown in Figure 9C, the immune-stimulating micelle (ACP-R837) could activate tumor-associated macrophages and promote the secretion of cytokines, leading to an augmented antitumor immune response. The chemotherapeutic micelle (PPP-DOX) could induce tumor cell death through chemotherapeutic toxicity.

### Multiple combination therapy

**Figure 10:**
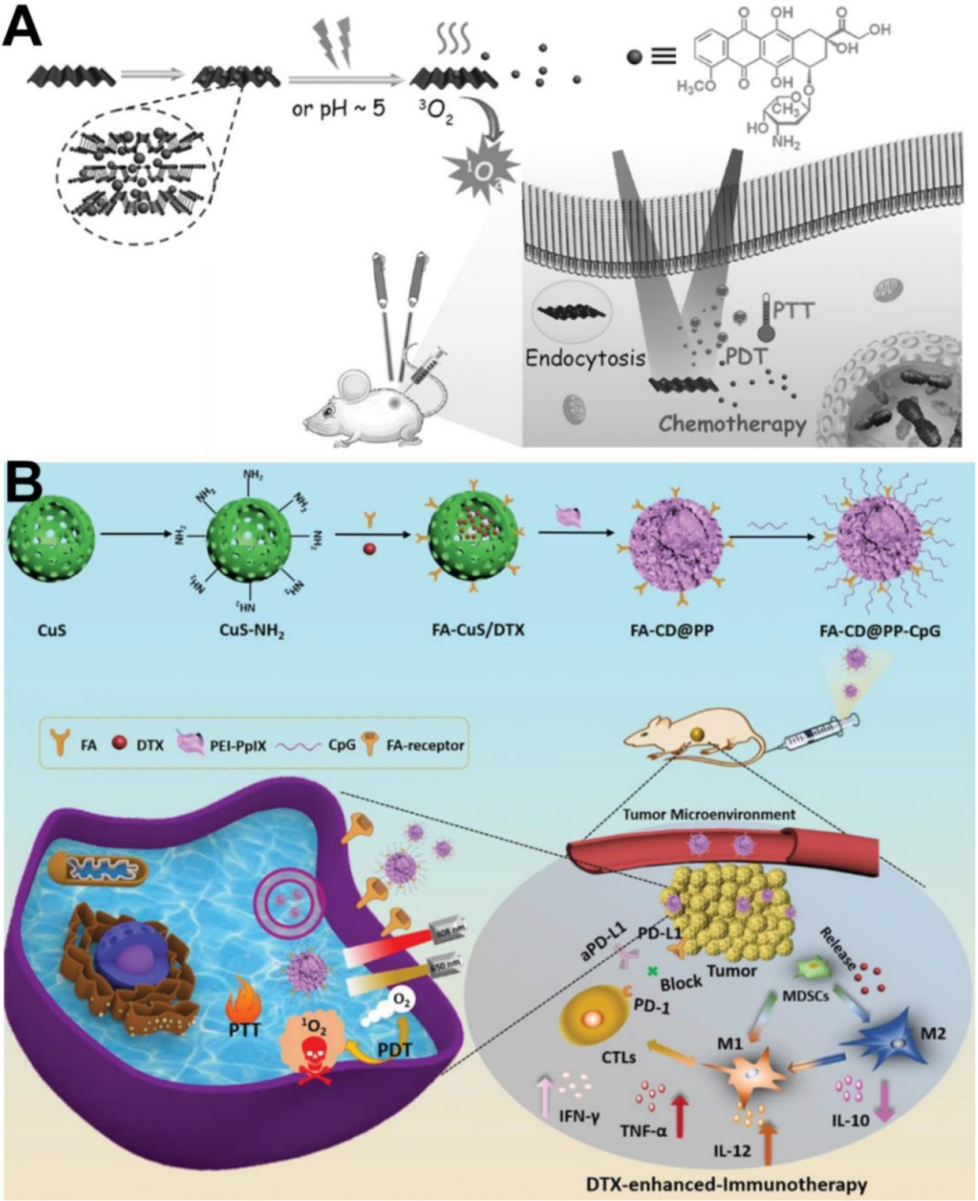
(A) Schematic illustration of BP-based drug delivery system for synergistic photodynamic/photothermal/chemotherapy of cancer 131. Copyright 2017, Wiley-VCH. (B) Rational design and synthesis of FA-CD@PP-CpG nanocomposites (top), its application in cancer treatment (left), and illustration of FA-CD@PP-CpG for docetaxel-enhanced immunotherapy (right) 133. Copyright 2019, Wiley-VCH.

To increase anticancer efficiency and tackle the difficulty of cancer recurrence, chemotherapy-based multi-therapy has been widely reported 129. Recently, the PTT/PDT/chemotherapy tri-modal anticancer strategy has been reported to improve therapeutic outcome and decrease drug dosage. For example, Li et al. 130 developed a multifunctional nanoplatform with PTT/PDT/chemotherapy tri-synergistic functions. This nanoplatform consisted of a pH-responsive diblock copolymer, Ce6, and the prodrug DOX and could reverse MDR in MCF-7/ADR tumor-bearing nude mice. Under NIR laser irradiation, Ce6 generated ROS for PDT and triggered the release of DOX for chemotherapy. The micelle could also efficiently convert NIR light to local heat for PTT and simultaneously enhance DOX penetration into tumors to improve the effectiveness of chemotherapy. In addition, because black phosphorus (BP) has both PTT and PDT effects, DOX-loaded BP can be used in multiple anticancer therapies *via* PDT/PTT/chemotherapy. For example, Guo et al. 131 reported an interesting concept of a BP nanosheet-based drug delivery system for synergistic photodynamic/photothermal/chemotherapy against cancer. In this system, BP not only effectively loaded higher amounts of DOX and showed pH-/photoresponsive drug release, but also generated ^1^O_2_ and possessed NIR photothermal activity (Figure 10A). Therefore, the tri-modal therapeutic system displayed dramatic therapeutic outcomes in 4T1-tumor bearing mice.

In addition, a PTT/PDT/chemotherapy/immunotherapy anticancer strategy has been reported. Jong et al. 132 reported BP flakes with plug-and-play and ultrasonic bubble bursting features, which can load DOX, PD-L1, and siRNA to fabricate BP-DcF@sPL nanoplatforms for chemo-photo-immunotherapy. *In vivo* antitumor results showed that ABP-DcF@sPL significantly inhibited tumor growth and prolonged the survival period in a C57BL/6 mouse model with MC-38 tumor xenografts. Similarly, metallic material-derived nanoparticles can be used as photosensitizers due to their PTT and PDT effects for cancer immunotherapy *via* ICD. For example, Dong et al. developed multifunctional FA-CuS/DTX@PEI-PpIX-CpG nanoparticles (named FA-CD@PP-CpG NPs) for synergistic PDT/PTT/chemotherapy/immunotherapy 133. As shown in Figure 10B, FA-CD@PP-CpG promoted infiltration of cytotoxic T lymphocytes (CTLs) to improve the efficacy of aPD-L1, suppressed myeloid-derived suppressor cells (MDSCs), and effectively polarized marrow-derived suppressor cells (MDSCs) toward the M1 phenotype to reduce tumor burden, further enhancing the antitumor efficacy. Therefore, FA-CD@PP-CpG nanocomposite is an efficient synergistic therapeutic modality in multi-combination therapy for anticancer treatment.

## Conclusions and future directions

Chemotherapy serves as one of the most important cancer treatment modalities and has been extensively used in clinic. However, conventional chemotherapeutics usually induce serious side effects due to their low half-lives in the blood and rapid distribution in healthy tissues and organs. Therefore, exploring and developing more efficient methods to enhance cancer chemotherapy is an urgent problem that must be solved. With the development of nanomedicine, multifunctional nanocarriers have showed a good application prospect in improving cancer chemotherapy. In this review, we systematically summarized different strategies to improve chemotherapy effects based on nanomedicine, including enhancement of chemotherapeutic drugs at tumor sites, chemotherapy towards specific subcellular organelles, reversal of resistance mechanisms, and combined chemotherapy. Despite the rapid development of nanotechnology and the development of multifunctional nanomedicines, the relevant research is still in its infancy. To reach clinical translation of research results as soon as possible, many key problems must be solved.

First, although NDDSs can improve enrichment of therapeutic drugs at tumors *via* passive targeting (EPR effect) or active targeting (modified with a targeting ligand), less than 0.7% of the injected dose of chemotherapeutic drugs accumulate in tumors 175, 176. The requirement of high accumulation in tumors seems to be a limiting factor in the development of nanomedicine. To address this bottleneck, a potent strategy will be to augment the permeability and retention of nanocarriers by developing NDDSs with step-by-step targeting capability, which can selectively increase vascular permeability, promote penetration of drug-loaded nanoparticles into tumor tissues, and target tumor cells and their subcellular organelles.

Second, assembling a reasonable combination of different treatment methods based on nanomedicine to achieve effective cancer treatment is still a challenge. Although combined chemotherapy has shown a significant therapeutic effect, related research is still in the early stage, and many important problems must be solved. For example, the mechanisms underlying combination therapy are still unclear or ambiguous. To deeply explain the mechanism of action of these combination therapies, it is necessary to select a variety of detection and analysis methods. In addition, because of tumor heterogeneity and differences in primary sites, the most reasonable combination strategies should be determined according to the different tumor types. Nanomedicine with diagnostic and therapeutic functions to achieve effective and successful cancer treatment can also be designed.

Third, although the reported nanomedicines have demonstrated excellent tumor therapeutic effects* in vivo* based on the current tumor models, pharmaceutical researchers and regulatory agencies recognize that results from preclinical animal models frequently fail to predict drug responses in humans 177, and thus, few of these nanomedicines have entered clinical studies. This actuality may be because animal models constructed for *in vivo* antitumor efficacy evaluation are not good substitutes for clinical tumor characteristics. Therefore, to make results more meaningful, it is important to explore and develop new animal models that are more consistent with human clinical characteristics. In future studies, humanized animal model may be a good choice and should be widely used in preclinical evaluation of nanomedicine. In addition, if possible, animal models of different species, especially large animals (*e.g.,* pigs and monkeys) that have immune systems similar to the human immune system, should be used for* in vivo* evaluation, so that the results can be more useful for clinical application.

Fourth, the safety of nanomedicine must be carefully considered. More studies on clinical safety, pharmacokinetics, biostability, and overall therapeutic effects should be intensively and extensively explored.

In summary, nanotechnology is an effective tool to enhance cancer chemotherapy, but there is still a long way to go, and more key challenges must be addressed before widespread clinical use can be achieved. More multidisciplinary and multiteam cooperation is required to promote clinical translation of nanomedicine-based cancer chemotherapy.

## Figures and Tables

**Table 1:** Clinically approved or under clinical trial nanomedicines

Product	Drug	Carrier components	Company	Stage	Ref.
Nab-Paclitaxel (Abraxane)	PTX	Human serum albumin	Abraxis BioScience	FDA and EMA approved	9
Genexol-PM	PTX	Micelle: mPEG-PDLLA	Samyang Biopharm	Approved in Korea	10
Apealea	PTX	Micelle: two isoforms of N-retinoyl-_L_-cysteic acid Methyl ester sodium salt	Oasmia Pharmaceutical	EMA approved	11
Lipusu	PTX	Liposome: lecithin/cholesterol	Nanjing Luye Sike Pharmaceutical Co.	Phase IV	12
Doxil	DOX	Liposome: HSPC, cholesterol, mPEG-DSPE	Johnson &Johnson	FDA and EMA approved	13
Myocet	DOX	Liposome: phosphatidylcholine, cholesterol	Teva	EMA approved	14
ThermoDox	DOX	Thermosensitive liposomal doxorubicin	MedKoo Biosciences Inc.	Phase III completed	15
Nanoparticle generator	DOX	Porous silicon microparticle with polymeric doxorubicin	/	Planning of phase I	16
NC-6004	Cisplatin	Micelle: PEG-P(Glu)	Nano Carrier Co.	Phase I/II	17
Lipoplatin	Cisplatin	Liposome: SPC/cholesterol/DPPG/mPEGDSPE	Regulon Inc.	Phase II/III	18
CRLX101	CPT	PEG-modified *β*-cyclodextrin	Cerulean Pharma Inc.	Phase II	19
NKTR-102	Irinotecan	PEG (four-arm) conjugation	Nektar Therapeutics	Phase II	20
Onivyde	Irinotecan	Liposome: DSPC, cholesterol, mPEG-DSPE	Merrimack Pharmaceuticals	Phase II/III	21
DOTAP: Chol-TUSC2	TUSC2	DOTAP: Chol	Genprex, Inc.	Phase I/II	22
Mepact	Mifamurtide	Liposome: POPC,OOPS	Takeda Pharmaceutical	EMA approved	23
Marqibo	Vincristine sulfate	Liposome: sphingomyelin, cholesterol	Talon Therapeutics	FDA approved	24
Vyxeos	Cytarabine and daunorubicin	Liposome: DSPC,DSPG, cholesterol	Jazz Pharmaceuticals	FDA and EMA approved	25

**Table 2:** Summary of chemotherapy-based combined cancer treatment

Combinational type	Agent regimen	Nanoplatform	Cancer type		Ref.
			*In vitro*	*In vivo*	
Combination with PDT	CPT + PtNP	CPT-TK-HPPH/Pt NP	CT26	CT26	134
	Cisplatin + Ce6	PCT@HCCT	4T1	4T1	68
	TPZ + porphyrinic MOFs	TPZ/UCSs	CT26	CT26	135
	CPT + PPa	MPEG-(TK-CPT)-PPa	HCT116	HCT116	136
	DOX + Ps	DOX-loaded; H-LTDC	HCT-116	HCT-116	101
	TPZ + ICG	iNP/IZ	4T1	4T1	103
	TPZ +Ce6	Lip/Ce6/TPZ-P_miRNA_	MCF-7	MCF-7	102
	TPZ +Ce6	TPZ/AI-NVs	HepG2	HepG2	138
Combination with PTT	SN38-Nif + ZrC NSs	ZrC@prodrug	A549 and SMMC-7721	SMMC-7721	139
	DOX + polyaniline	PANI-ES@AOT-V-D	MCF-7 and Hela	MCF-7	140
	DXL + AuNPs	Au/Fe_3_O_4_/PVA-10%DXL	MCF-7	MCF-7	141
	DOX + PDA	F@PDA-TPP/SS/DOX	B16F10	B16F10	106
	DOX + Bi_2_Se_3_	Bi_2_Se_3_/DOX@MPs	H22	H22	142
	DOX + iron oxide nanoparticles	DOX/MNP-PMs	A549	/	143
	DOX + MPN	MSN@MPN@DOX	A549	/	144
	DOX + B nanosheets	DOX-17AAG@B-PEG-cRGD	MDA-MB-231	MDA-MB-231	145
	DOX + Cu_2_-xSe	PT-V@TPDOX	MCF-7 and MCF-7/ADR	MCF-7/ADR	72
	Pt(IV) + IR780	Pt-I-IR780 NPs	4T1	4T1	146
	Tyroservatide + PpIX	PpIX NAs	4T1, MCF-7, A2780/Taxol and MCF-7/DOX	4T1	147
	DOX/TAX + PpIX	PM-DOX-TAX	MDA-MB-231	MDA-MB-231	105
	DOX + IR820	LA-IR820/DOX ND	HepG2	/	148
Combination with CDT	TMZ + MnO	iRPPA@TMZ/MnO	C6	C6	149
	Cisplatin + Fe(III)	PtH@FeP	4T1	4T1	113
	TPZ + Fe(III)	HGTFT	4T1	4T1	150
	DOX + Cu (Ⅱ)	DOX@Cu_2_O-PEG NCs	MCF-7	MCF-7	151
	Cisplatin + EGCG	PTCG NPs	HepG2	HepG2	112
	DOX + MIL-100	DMH NPs	MCF-7	MCF-7	108
	Platinum(IV) + MnO_2_	UCMnPt	HepG2	HepG2	152
	DOX + MnO_2_	AMSNs/DOX	Huh7	Huh7	153
	CPT + MnO_2_	MS@MnO_2_-CPT	U87MG	U87MG	154
	CPT + Iron oxide NPs	LaCIONPs	A549	A549	155
	platinum(IV) + Fe^3+^	Fe-DSCP-PEG-cRGD	C6	C6	156
	DOX + PB	DOX-Au@PB	SMMC-7721	SMMC-7721	157
	DOX + copper/iron(II)	Cu-Fe-MSNs-DOX	Hela	/	158
	DOX+ MnO_2_	MnO_2_@HMCuS-DOX	MCF-7	/	159
	Cisplatin + Fe^2/3+^	Pt&Fe_3_O_4_@PP	U87 MG	U87 MG	160
	Vitamin k3 + Cu^2+^	Vk3@MOF-199 NPs	4T1	4T1	161
Combination with radiotherapy	DSP + Hf	BM@NCP(DSP)-PEG	4T1	4T1	114
Combination with gas therapy	PTX + S-nitrosoalbumin	Ptx@AlbSNO	4T1	4T1	115
	DOX + NTC	NO-M@DOX	MCF-7/ADR	MCF-7/ADR	162
	DOX + GSNO	DOX@GSNO-HAPNs	MCF-7	MCF-7	163
	DOX + DEA/NO	DOX@HA-DNB-DEA/NO	SMMC-772	SMMC-772	164
Combination with immunotherapy	CUR + NLG919	PEG-CDM-PEI-P(CURDT)	B16F10	B16F10	126
	DOX + R837	PPP-DOX + ACP-R837	4T1	4T1	127
	DTX + GM-CSF	gETL NPs	CT26	CT26	165
	DOX + JQ1	IRG@JQ1/DOX	4T1	4T1	166
	SF + M1-type macrophages	M1/SLNPs	Hepa1-6	Hepa1-6	167
	DOX + siRNA	PEG@D:siRNA	CT26	CT26	124
	DOX + anti-PD-L1	CM@MON@DOX	4T1	4T1	168
	PTX + anti-PD-1	sAMcP	B16F10	B16F10	125
	Oxaliplatin + ApoA1 mimic peptide	TA-OBL	4T1	4T1	169
	DOX +CpG	LMWH/PPD/CpG	B16F10	B16F10	127
	GEM + 1MT	GEM-1MT NPs	B16F10	B16F10	170
Multiple combination therapy	SN38 + PDA + PZM	FA-PPSM	Eca-109	Eca-109	171
	DOX + γ-radiation + Seleninic acid	PSeR/DOX NPs	MDA-MB-231	MDA-MB-231	172
	PTX + IR780 + S-nitrosothiol	HSA-NO	4T1	4T1	173
	DOX + Ce6	PDPC	MCF-7/ADR	MCF-7/ADR	130
	DOX + BP	BP-DOX	4T1	4T1	131
	DOX + BP + aPPL	BP-DcF + aPL	MC-38	MC-38	132
	DTX + PpIX + CpG	FA-CD@PP-CpG	4T1	4T1	133
	R837 + IR820 + 1MT	HA/IR820@ZIF-8 + MAN/(R837+1 MT)@ZIF-8	B16F10	B16F10	174
